# Comparing traditional audits with electronic measurements: bringing a new perspective to measurement of compliance with hand hygiene protocols

**DOI:** 10.1186/2047-2994-4-S1-P300

**Published:** 2015-06-16

**Authors:** J Hayhurst, H Gentry, J Kirk, J Arbogast, C Galani, AN Eaton

**Affiliations:** 1 GOJO INDUSTRIES - EUROPE Ltd, Milton Keynes, UK; 2 Worcestershire Acute Hospitals NHS Trust, Worcester, UK; 3 GOJO INDUSTRIES Inc., Akron OH, USA

## Introduction

Manual auditing has long been considered as the gold standard for measuring concordance with hand hygiene protocol. Until recently, it could be argued that it was the only valid methodology available. This is despite the fact that self-auditing has long been deemed to be unreliable, and even “Secret Shopper” audits have been found to produce biased results due to variances in the methodology used and to the ‘Hawthorne Effect’[1].

In the UK NHS, compliance rates >90% are widely reported [2], leading Trust management to believe that hand hygiene is under control and no additional time or money will be spent improving practice.

Electronic monitoring systems are available, which due to their different methodology, provide alternative yet complementary results.

## Objectives

To compare results reported to Trust Management and audits to those obtained from ECM to understand how they interrelate and to gain new insights into hand hygiene compliance.

## Methods

Trust results and “Secret Shopper” audit results were compared against the ECM data, and audits recorded using a smartphone-based system that feeds results directly to the ECM. This allows a comparison between the Wash-in, Wash-out methodology of the ECM and the 5-moments of the long-duration manual audits.

## Results

Short-duration audits were slightly lower than the >90% reported to Trust Management. ECM results were found to be significantly lower, typically <20%.

## Conclusion

By using a different methodology, ECM offers a different perspective on the issue of hand hygiene compliance. Whilst it will never replace manual auditing, it does provide an alternative view to the rose-tinted compliance figures that are often used and provides unique data that can be used in training / education, evaluation and feedback, and as reminders in the workplace, as part of the WHO Multimodal Hand Hygiene Improvement Strategy.

## Disclosure of interest

None declared.
